# Transcriptomic and metabolomic landscape of quinoa during seed germination

**DOI:** 10.1186/s12870-022-03621-w

**Published:** 2022-05-10

**Authors:** Yuqiong Hao, Yechun Hong, Huimin Guo, Peiyou Qin, Ancheng Huang, Xiushi Yang, Guixing Ren

**Affiliations:** 1 Institute of Crop Sciences, Chinese Academy of Agricultural Sciences, No. 80 South Xueyuan Road, Haidian District, Beijing, 100081 China; 2 Key Laboratory of Molecular Design for Plant Cell Factory of Guangdong Higher Education Institutes, Department of Biology, SUSTech-PKU Institute of Plant and Food Science, Southern University of Science and Technology, Shenzhen, 518055 Guangdong China; 3 Biotechnology Research Institute, Shanghai Academy of Agricultural Sciences, Shanghai, 201106 China; 4 Institute of Bast Fiber Crops, Chinese Academy of Agricultural Sciences, Changsha, 410205 China; 5 College of Pharmacy and Biological Engineering, Chengdu University, No. 1 Shilling Road, Chenglo Avenue, Longquan District, Chengdu, 610106 China

**Keywords:** *Chenopodium quinoa*, Seed germination, Transcriptome and metabolome, Phytohormone, Starch and sucrose metabolism

## Abstract

**Background:**

Quinoa (*Chenopodium quinoa*), a dicotyledonous species native to Andean region, is an emerging crop worldwide nowadays due to its high nutritional value and resistance to extreme abiotic stresses. Although it is well known that seed germination is an important and multiple physiological process, the network regulation of quinoa seed germination is largely unknown.

**Results:**

Here, we performed transcriptomic study in five stages during transition from quinoa dry seed to seedling. Together with the GC–MS based metabolome analysis, we found that seed metabolism is reprogrammed with significant alteration of multiple phytohormones (especially abscisic acid) and other nutrients during the elongation of radicels. Cell-wall remodeling is another main active process happening in the early period of quinoa seed germination. Photosynthesis was fully activated at the final stage, promoting the biosynthesis of amino acids and protein to allow seedling growth. The multi-omics analysis revealed global changes in metabolic pathways and phenotype during quinoa seed germination.

**Conclusion:**

The transcriptomic and metabolomic landscape depicted here pave ways for further gene function elucidation and quinoa development in the future.

**Supplementary Information:**

The online version contains supplementary material available at 10.1186/s12870-022-03621-w.

## Introduction

Seed germination is an important process that brings seed plant into natural and agricultural eco-system. The process has attracted much attention in a long period, not only for the secret in this life cycle transition, but also for its impacts on production and quality for crops [1, 2]. Generally, dry seed begins to germinate when encountering water under a favorable condition, followed by the activation in physiology, morphology, and biochemistry, etc. This first step so-called water imbibition during seed germination can be further divided into three phases, i.e., fast water uptake, metabolism reprogramming, and radicle emergence [3]. The early stages of seed germination are crucial process that broke the ‘low-hydrated’ and “weak metabolic” of dry seed, and the complex processes involved in this development event include DNA damage and repairing, phytohormone metabolism and signal transduction, nutrient and energy metabolism, cell wall remodeling and modification, etc. [4]. Followed by the reactivation in many aspects and processes, the radicles continue to elongate and cotyledon appears and expands in post-germination stage, during which photosynthesis and energy metabolism associating processes promote the final seedling establishment [5]. The molecular mechanism underlining the dynamics of seed germination is important for seed biology that warrants investigation.

Quinoa (*Chenopodium quinoa* Willd.), a dicotyledonous plant native to the Andean region, belongs to the Chenopodiaceae family together with spinach (*Spinacia oleracea L.*) and sugar beet (*Beta vulgaris L.*) [6]. Quinoa is becoming popular to global market for its capability to fulfilling nutrient requirement like protein, unsaturated fatty acids and vitamins [7]. It has been reported that quinoa proteins have a balanced amino acid composition and multiple functional activities such as cholesterol-lowering and α-glycosidase inhibitory activities. Quinoa is rich in high-quality protein, healthy oil, saponins, polyphenols, and flavonoids, thereby qualifying for consumers’ healthy diet [8]. To raise interest in this ancient and special crop, the year 2013 has been declared as the “International Year of Quinoa” by The Food and Agriculture Organization of the United Nations (FAO) [9]. Meanwhile, quinoa is found to exhibit strong tolerance to extreme climate and stress conditions like drought and high salinity, which make it more interesting in agriculture to deal with global warming and food limitation [10, 11].

During seed germination, the nutritional composition of quinoa is altered by activation of complex processes involved in protein synthesis and storage [12]. However, the molecular dynamics behind quinoa seed germination is still largely unknown, since most present studies on quinoa mainly focus on characterization of nutrition and chemical composition [13]. The releases of quinoa genome sequence provided the most valuable resource for molecular study, together with the rapid development of integrated-omics studies including transcriptomics and metabolomics analysis [14–16]. Here, to explore the dynamics during quinoa seed germination and the corresponding regulatory network, comprehensive transcriptome and metabolome profiling with focus on phytohormones signal transduction, storage reserves metabolism, and nutrition mobilization were performed. Our work yielded insights into the complex processes during quinoa seed germination, which is of benefit for future quinoa improvement.

## Results and discussion

### Global analysis of dynamic changes during quinoa seed germination by transcriptomic and metabolic profiling

**Fig. 1:**
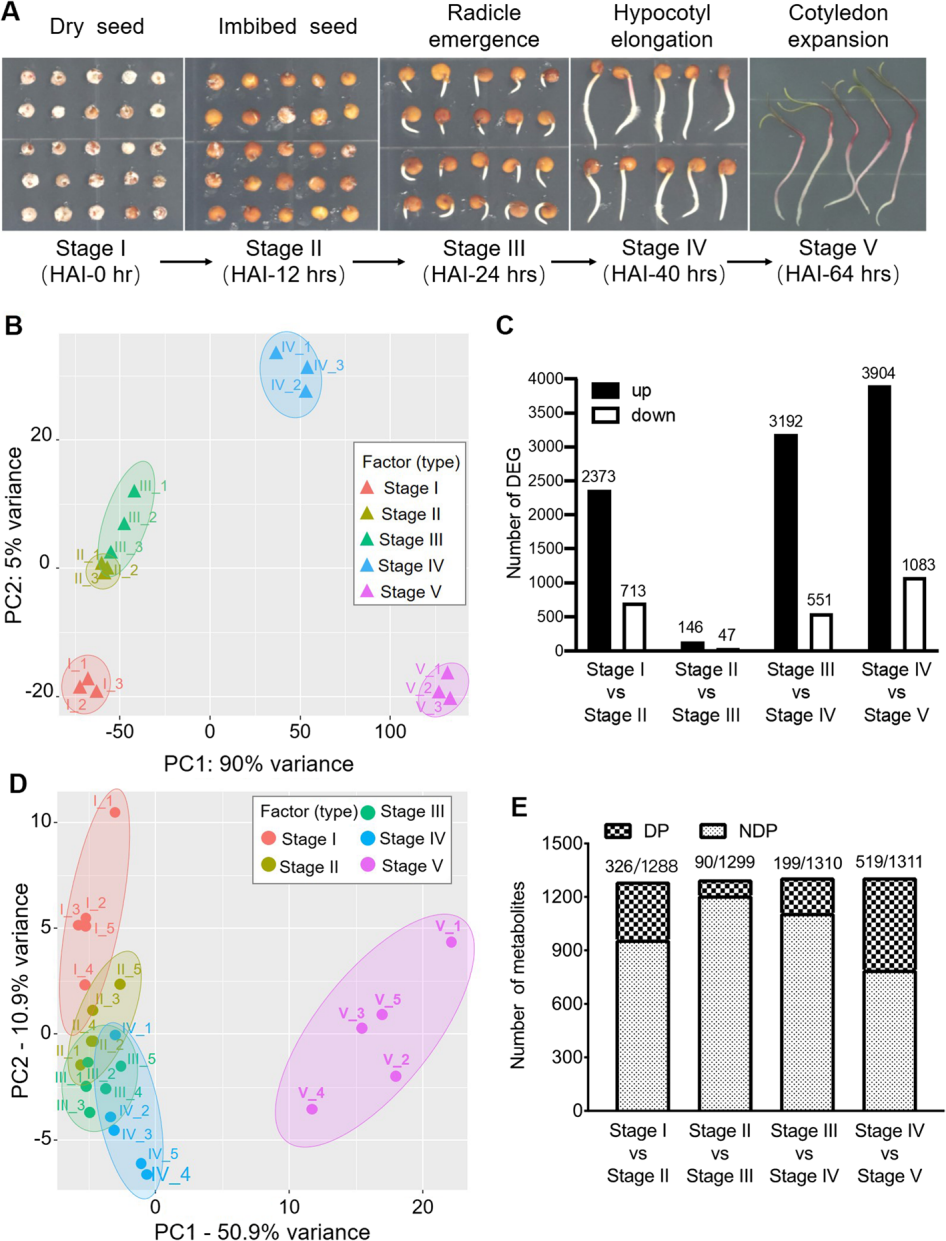
Transcriptomic and metabolic analysis reveal dynamic transition during seed germination of quinoa. **A** The five stages of transitions during quinoa germination from dry seed to seedling. The pictures representing each stage were taken at indicated hours after seed imbibition. HAI, hours after imbibition. **B** Principal component analysis (PCA) of transcriptome data obtained from five stages samples during quinoa seed germination. **C** Numbers of up and down-regulated genes during transition of germination stages. **D** PCA of GC–MS profiling identified the metabolic changes within five stages transition during quinoa seed germination. **E** Numbers of differential metabolites during transition of germination stages. DP, differential peaks. NDP, non-differential peaks

For plant, seed germination is a critical event in life cycle which is comprised of multiple steps. To better understand physiological changes and establish the platform for further omics studies, we performed the seed germination assay in quinoa dry seeds, and the dry seed was designed as the first stage in our experiment. Seed germination begins with a rapid water uptake indicating by increase of fresh weight at first 12 h after seed imbibition, and ends up with the protrusion of radical. The seed size along with the fresh weight continuously increased, promoting radical emergence in the germination process (Fig. 1A and Fig S1A). Following the emergence of quinoa seed radical, hypocotyl was elongated to next stage. Subsequently, cotyledons gradually unfolded to facilitate the establishment and growth of green seedling (Fig. 1A). Quinoa germination was completed by visible radicle protrusion through the testa, and followed by post-germination stage (hypocotyl elongation and cotyledon expansion) for seedling establishment. Based on the physiological and morphological divergence during this dynamic process, we collected quinoa samples at distinct five stages including dry seed (stage I), imbibed seed (stage II), radical emergence (stage III), hypocotyl elongation (stage IV) and cotyledon expansion (stage V), and performed omic studies to study molecular dynamics behind (Fig. 1A).

To identify key genes involved in regulating quinoa seed germination and transition to seedling establishment, we firstly carried out RNA-seq analysis in above five stages samples. After filtering out low-quality reads, we obtain a transcriptomic data with high quality with average 20.65 million paired-end clean reads, among ~ 90% of which successfully mapped to reference genome. More than 35,000 expressed transcripts were obtained based on our transcriptome data and we further used customed R scripts to annotate all DEGs with genome annotation files ‘Cquinoa_392_v1.0.annotation_info.txt’ from the JGI databases. The GC contents (43.63%-47.49%) and high Q30 value (92.02%-94.93%) enabled the next differential expression analysis (Table S1). Firstly, principal component analysis (PCA) was performed and showed the distribution of the five stages expect for a partial overlap between stage II and III (Fig. 1B), and a high similarity was found in biological replicates. Secondly, differentially expressed genes (DEGs) were screened based on two aspects of log_2_(fold change) and statistical significance level (adjusted *P*-value, Table S2), and a Venn diagram showed the identified DEGs between any two neighboring stages, indicating the stage specificity (Fig S1B). Finally, we counted the numbers of up- or down-DEGs by comparation between any two neighboring stages. Thousands of up- or down-regulated genes are identified between different stages during seed germination indicates specificity of each stage, while only a small number of DEGs identified in stage II versus stage III suggesting the similarity of the two stages (Fig. 1C).

In order to better explore the dynamics from quinoa dry seed to seedling in metabolic level, we next perform a metabolomic profiling analysis by Gas Chromatography-Mass Spectrometry (GC–MS) in the five distinct stages samples. PCA was conducted and revealed a significant separation among the different stages, indicating the metabolic changes occurred in transition of seed germination (Fig. 1D). The number of detected metabolite peaks in each stage and differentially metabolites in any two stages were counted after processing of the raw GC–MS data (Fig. 1E). A Venn diagram showed the differentially metabolites between any two neighboring stages indicated the stage specificity (Fig S2A). The significantly differentially metabolites were imported into KEGG enriched analysis to generate the metabolome view (Fig S2B). KEGG analysis of differentially metabolites between neighboring stages exhibited largely difference, provides important information with transcriptome data in further exploration of dynamics of quinoa seed germination.

### Phytohormones signals play important roles in early quinoa seed germination

**Fig. 2:**
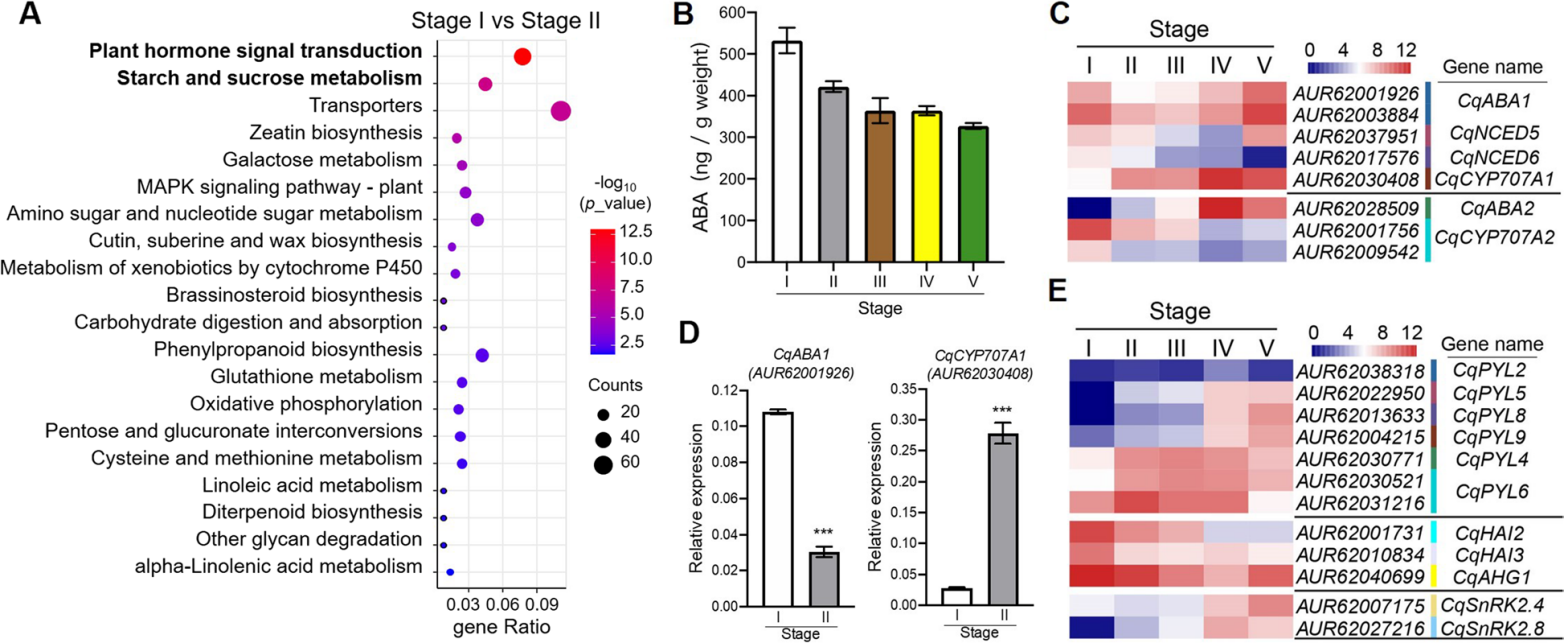
Abscisic acid (ABA) metabolism was activated during the early stages of quinoa seed germination. **A** KEGG analysis of DEGs identified between the first two stages verified the important roles of plant hormone signals in transition from dry seeds to imbibed seeds (www.kegg.jp/kegg/kegg1.html). **B** Detection of abscisic acid (ABA) contents in germinating seeds of different stages. **C** Heatmap analysis of DEGs involved in biosynthesis and catabolism of ABA. The important components in ABA biosynthetic pathway contain *CqZEP*/*ABA1*, *CqNCEDs* and *CqABA2*. Catabolism of ABA was mediated by *CqCYP707As* encoding cytochrome P450 monooxygenase. **D** qRT-PCR analysis of an ABA biosynthetic gene *CqABA1* and an ABA catabolism associated gene *CqCYP707A1* in first two stages of quinoa seed germination. Values are means ± SD (*n* = 3). *** *P* < 0.001, Student's t-test. **E** Heatmap analysis of DEGs involved in ABA core signaling transduction during quinoa seed germination. *CqPYLs* encoding ABA receptors, *CqHAIs* / *CqAHG1* encoding PP2C protein phosphatases, and *CqSnRK2s* encoding SnRK2 protein kinases

To understand the global expression patterns during quinoa seed germination, we tried to map the processes by functional annotating the DEGs against the Kyoto Encyclopedia of Genes and Genomes (KEGG) database. In this study, the KEGG analysis of DEGs between neighboring stages exhibited largely difference, suggesting multiple stage specific processes arose during the rapid transition of quinoa seed germination (Fig S1C). Within the KEGG analysis between Stage I versus II, the first period that dry seed began to germinate, “plant hormone signal transduction” was significantly enriched, following by other processes such as “starch and sucrose metabolism” (Fig. 2A). It has been well demonstrated that abscisic acid (ABA) plays a central role in regulating seed germination in plant [17], therefore we firstly analyzed the content of ABA in germinating quinoa seeds by enzyme linked immunosorbent assay (ELISA), and the result showed that dry seed (stage I) obtained a highest level of ABA, and significantly decreased in the stages afterward during germination (Fig. 2B), suggesting that ABA may play a conserved role in inhibiting seed germination as in other plant species. According to the genetic and functional studies in *Arabidopsis*, the important components in ABA biosynthetic pathway contain *ZEP*/*ABA1* encoding zeaxanthin epoxidase which catalyzes zeaxanthin to all-trans-violaxanthin, *NCED*s encoding 9-cis-epoxycarotenoid dioxygenase that oxidase both 9′-cis-neoxanthin and 9′-cis-violaxanthin, and cytosolic *ABA2* and *AAO3* encoding short-chain alcohol dehydrogenase and abscisic aldehyde oxidase respectively to convert xanthoxin into ABA [18, 19]. Catabolism of ABA mediated by *CYP707As* encoding cytochrome P450 monooxygenase also determines the ABA level [20]. We used a heatmap analysis to see the transcript pattern of DEGs involved in ABA metabolism, several genes annotated as *CqABA1*, *CqNCED5* and *CqNCED6* display a higher expression level in dry seed comparing to germinating seed, though an increase of transcript level of *CqABA1* and *CqNCED5* were observed in final stage during seed germination (Fig. 2C, Table S3). Besides, *CqCYP707A1* exhibited a lower expression level in dry seed but increased in the after stages, which may contribute to the decrease of ABA content during seed germination (Fig. 2C, Table S3). Other components like *CqABA2*, *CqCYP707A2* possibly have distinct functions in regulating ABA metabolism based on their different expression patterns (Fig. 2C, Table S3), which is consistent with previous study that showed *AtCYP707A* genes played differential roles in regulating seed germination in *Arabidopsis* [21]. Among these genes, the transcript levels of *CqABA1* (*AUR62001926*) and *CqCYP707A1* (*AUR62030408*) were further confirmed by qRT-PCR analysis (Fig. 2D). The down-regulation of *CqABA1* and up-regulation of *CqCYP707A1* contributed to the decrease of ABA content during quinoa early germination. Finally, we identified the DEGs encoding ABA signaling core components, *CqPYLs* encoding ABA receptors, *CqHAIs* / *CqAHG1* encoding PP2C protein phosphatases, and *CqSnRK2s* encoding SnRK2 protein kinases. The relative low transcript levels of *CqPYLs* and high level of *CqPP2C* suggesting that the ABA signaling pathway tends to be silenced in dry seed although a high content of ABA in seeds. Moreover, the differential of expression level or patterns of *CqPYLs* suggesting the diversity of *PYLs*, which was consistent with cases in other plant species (Fig. 2E, Table S3) [22, 23].

Besides ABA, we also analyzed DEGs associated with other plant hormones, including gibberellin (GA) (Fig S3A), auxin (Fig S3B) and cytokinin (Fig S3C). Gibberellin is another central hormone that has been widely recognized in promoting seed germination [24]. The transcript levels of genes participating in gibberellin biosynthetic pathway like *CqGA*_*20*_*OX* and *CqGA*_*3*_*OX* and gibberellin receptor *CqGID1* were significantly up-regulated in the stage of radicle emergence, indicated an important role of GA in promoting seed germination (Fig S3A). A cluster of genes involved in other hormones like auxin and cytokinin were also enriched, such as auxin related *IAA* and *GH3* family genes, cytokinin synthase and ARR regulators, suggesting these hormones possibly play functions in regulating quinoa seed germination though it was not thought as important as ABA or GA in this process (Fig S3). Among these, six *GH3* genes encoding indole-3-acetic acid-amido synthetase were significantly up-regulated at germinating and post-germination stages, which possibly maintained auxin homeostasis during quinoa seed germination according to studies toward their homolog in *Arabidopsis* [25]. The increase of GH3 proteins functioning in conjugating excess IAA to various amino acids may be the case in quinoa to maintain the dynamic balance of auxin for quinoa germination and growth. These results indicate that ABA and GA signaling and their potential integration with other hormonal signaling are required and essential during seed germination in quinoa.

### Nutrient and energy metabolism was activated during quinoa germination

**Fig. 3:**
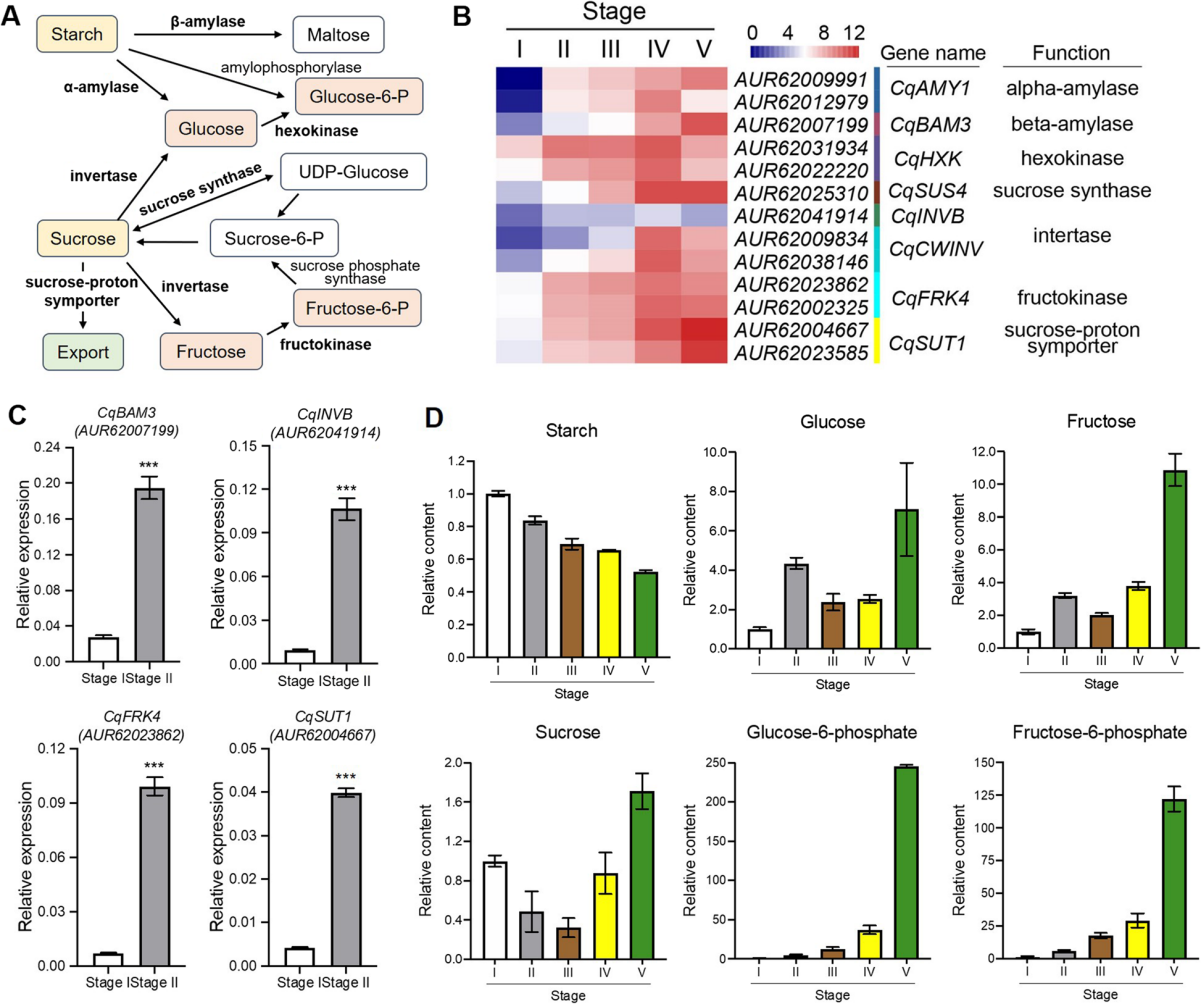
Starch and sucrose metabolism was activated during quinoa seed germination. **A** The model and key components of starch and sucrose metabolism in plant. The highlight boxes indicated the metabolites quantified from the GC–MS results. Glucose-6-P, glucose-6-phosphate. Sucrose-6-P, sucrose-6-phosphate. Fructose-6-P, fructose-6-phosphate. **B** Heatmap analysis of DEGs involved in starch and sucrose metabolism. **C** qRT-PCR analysis of three genes encoding enzymes involved in starch and sucrose metabolism, *CqINVB*, *CqBAM3* and *CqFRK4,* and *CqSUT1* encoding sucrose-proton symporter in first two stages of quinoa seed germination. Values are means ± SD (*n* = 3). *** *P* < 0.001, Student's t-test. **D** Analysis of changes of the represented metabolites in starch and sucrose metabolism during quinoa seed germination, including starch, sucrose, glucose, glucose-6-P, fructose and fructose-6-P. Values are means of three independent experiments ± SD, indicated the relative content of target metabolites based on the peak area

Metabolic activities in dormant seeds are weak or silenced until seed imbibition occurs to broke dormancy and began seed germination [26]. Once the germination was activated, seed stored reserves including starch, sucrose, proteins, and lipids are mobilized during the transition to enable seedling establishment, and starch and sucrose metabolism likely happened in the early stages during quinoa seed germination to provide nutrient and energy (Fig. 2A). In order to identify important genes functioning in promoting seed germinating, we searched the major components in starch and sucrose metabolism pathway (Fig. 3A). Genes annotated as *CqAMY1* and *CqBAM3* encoding alpha- and beta-amylase respectively were identified and showed significantly increasing after seed germinating, indicating their roles in leading starch converse to glucose and maltose respectively (Fig. 3B, Table S3). Three invertase candidates that degraded sucrose to glucose and fructose displayed similar pattern, and the up-regulated transcript level of *CqSUT*s encoding sucrose-proton symporter suggesting that exportation of sucrose is also activated in the beginning of seed germination, since sucrose is participating in other process and function in the normal energy provision (Fig. 3B, Table S3). The increase of transcript levels of *CqBAM3* (*AUR62007199*)*, CqINVB* (*AUR62041914*), *CqFRK4* (*AUR62023862*), and *CqSUT1* (*AUR62004667*) were further verified by qRT-PCR assay (Fig. 3C). Consistent with expression data, starch content decreased gradually during germination, and sucrose content changed rapidly, used for energy provision in time (Fig. 3D). Glucose and fructose content were significantly enhanced at the imbibed seeds, glucose-6-phosphate and fructose-6-phosphate content were continues increased during quinoa germination, used for glycolysis and tricarboxylic acid (TCA) cycle metabolism (Fig. 3D). The result demonstrated that starch and sucrose metabolism provided the main energy at the very early stages during quinoa seed germination.

### Remodeling and modification of cell walls are important during seed germination

To further study the molecular dynamics occurring during quinoa seed germination, Gene ontology (GO) analysis was conducted, and cell-wall remodeling and modification associating processes were enriched in the intervals of dry seeds versus seeds imbibition (Fig S4). Within this GO analysis, cell wall remodeling associated processes including “glucan metabolic process” and “cellular glucan metabolic process” in biological process group, together with “xyloglucan-xyloglucosyl transferase activity” and “transferase activity” in molecular function group, as well as “cell wall” in cellular components group were significantly enriched (Fig S4). Plant cell walls are composed of complex matrices including cellulose, glucans, hemicelluloses and pectins, which provide the mechanical properties of cell and tissue [27]. Besides, the primary walls controls cell growth and contributes to the energy metabolism for its high carbon fixation, so the cell-wall recycling by degradation, reorganization, and modification is important during fast growth phase such as seed germination [28].

**Fig. 4:**
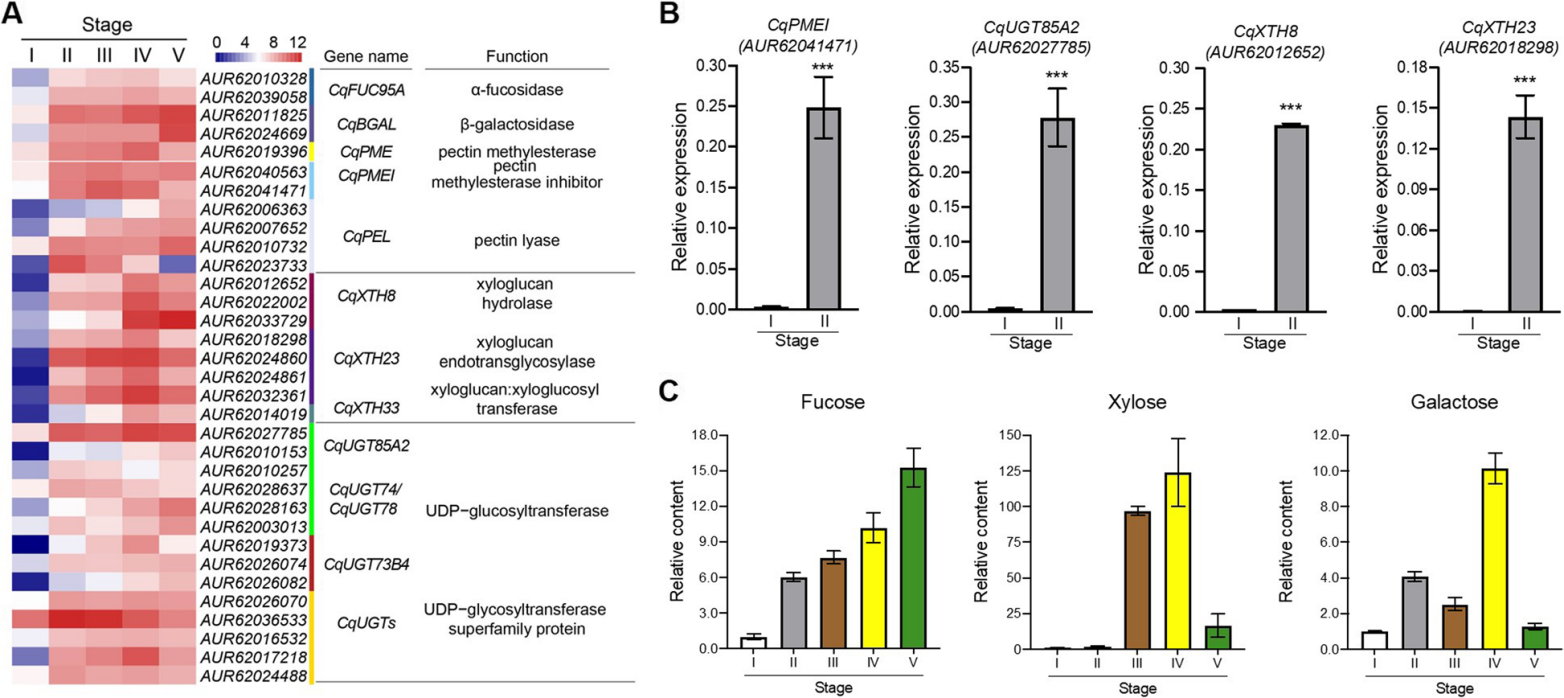
Quinoa seed germination was accompanied by remodeling and modification of cell wall. **A** Heatmap analysis of DEGs involved in cell wall remodeling and modification process during quinoa seed germination. α-fucosidase and β-galactosidase are important enzymes in xyloglucans degradation, and enzymes like PME, PMEI and PEL are involved in pectin degradation. Genes encoding xyloglucan hydrolase, xyloglucan endotransglycosylase, and xyloglucan:xyloglucosyl transferase are associated with cellular xyloglucan metabolic process, catalyzing the remodeling and mobilization of xyloglucan chains. UDP-glycosyltransferases, catalyzed the formation of glycosidic bonds by using nucleotide sugars as monosaccharide donor, promoting the synthesis of cell wall polysaccharides. The genes associated in above processes are significantly up-regulated after seed imbibition. **B** The transcript levels of four genes involved in cell-wall remodeling, *CqPMEI*, *CqXTH8*, *CqXTH23* and *CqUGT85A2*, were detected by qRT-PCR assay, which were upregulated at imbibed stage during germination. Values are means ± SD (*n* = 3). *** *P* < 0.001, Student's t-test. **C** Dynamic changes of some metabolites involved in cell wall synthesis and degradation, including fucose, galactose, and xylose. Values are means of three independent experiments ± SD, indicated the relative content of target metabolites based on the peak area

In order to better verify the molecular regulation of cell-wall remodeling during quinoa seed germination, we examined the DEGs involved in the multiple processes including genes functioning in degradation of primary cell wall. DEGs annotated as *CqFUC95A* encoding α-fucosidase and *CqBGAL* encoding β-galactosidase are significantly up-regulated right after seed imbibition, which has been characterized as xyloglucans degradation associating enzymes in *arabidopsis* (Fig. 4A, Table S3). Another cluster of DEGs related to pectin degradation shows similar pattern during this transition, including genes encoding pectin methylesterase (*PME*), pectin methylesterase inhibiter (*PMEI*) and pectin lyase (*PEL*) (Fig. 4A, Table S3). Besides, genes associated with cellular xyloglucan metabolic such as xyloglucan hydrolase, xyloglucan endotransglycosylase, and xyloglucan:xyloglucosyl transferase were highly expressed within the intervals of radicle emergence, which catalyze the remodeling and mobilization of xyloglucan chains in hemicellulose, weaken cellulose microfibrils, promote radical protrusion (Fig. 4A, Table S3) [29]. Genes encoding UDP-glycosyltransferases (*CqUGTs*), catalyzed the formation of glycosidic bonds by using nucleotide sugars as monosaccharide donor, promoting the synthesis of cell wall polysaccharides, was also significantly enriched within the intervals of radicle emergence (Fig. 4A, Table S3) [30]. These result from global transcriptomic aspect demonstrated cell-wall remodeling processes are significantly active, contributing to the rapid transition of early seed germination.

According to further confirm to transcriptomic analysis associating with cell-wall remodeling, four related genes including *CqPMEI* (*AUR62041471*), *CqUGT85A2* (*AUR62027785*), *CqXTH8* (*AUR62012652*) and *CqXTH23* (*AUR62018298*) were chosen for qRT-PCR verification, which showed significantly up-regulated at the imbibed seeds (Fig. 4B). Consistent with expression data, our metabolic profiling analysis showed cell wall sugars are increased during seed germination, such as fucose, galactose and xylose (Fig. 4C). The dynamic change of these metabolites suggested that activation of cell-wall remodeling pathway not only speeds the construction of cell wall but also participates in metabolic recycling associating with energy metabolism and multiple primary and secondary metabolism (Fig S1B).

### Photosynthesis process was highly activated after the stage of hypocotyl elongation

**Fig. 5:**
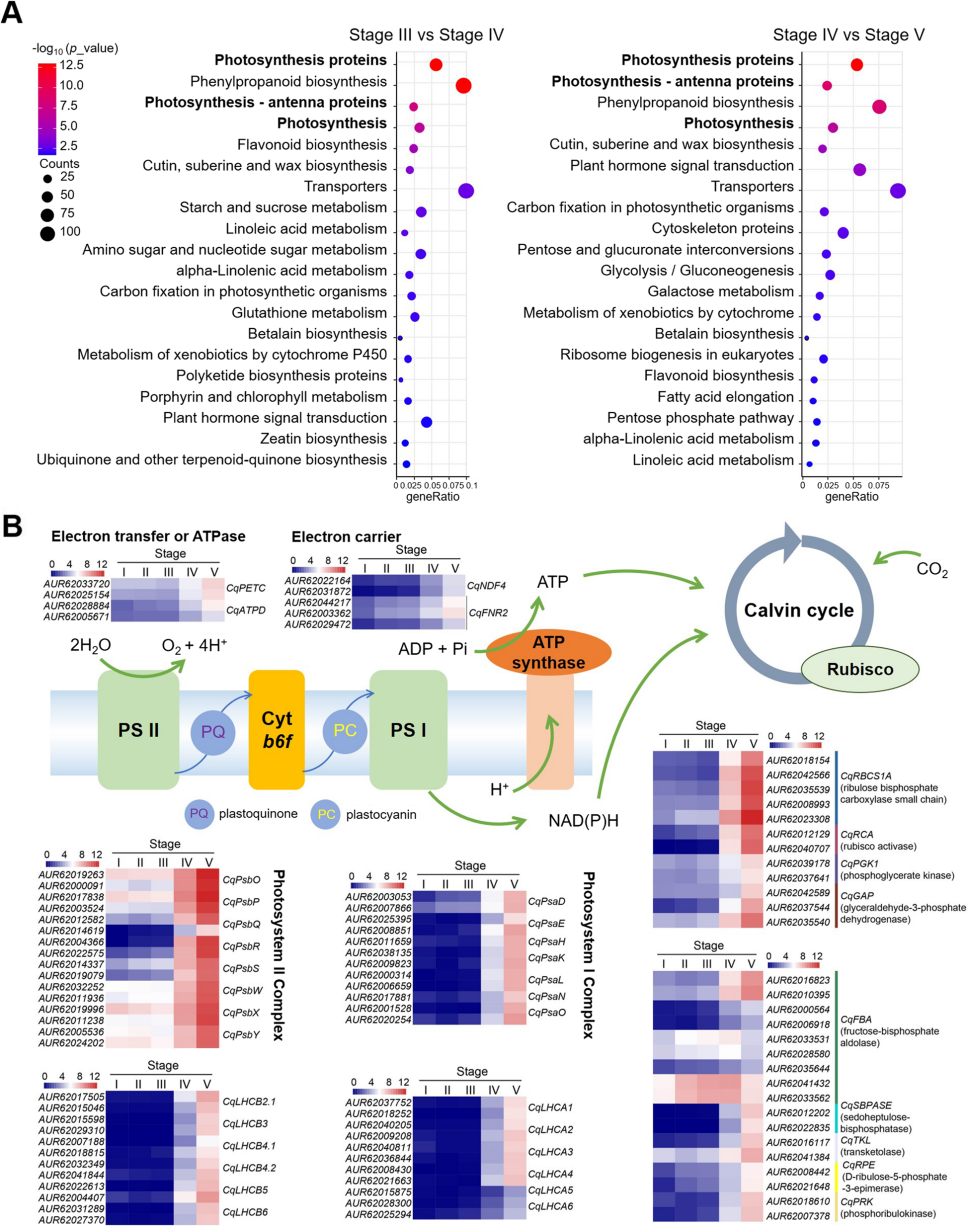
Photosynthesis processes were initiated at late stages of quinoa seed germination. **A** KEGG analysis of DEGs identified between stages III and IV (radicle emergence vs hypocotyl elongation, left), and stages IV and V (hypocotyl elongation vs cotyledon expansion, right), both indicated photosynthesis as one key process in accomplishment of quinoa seed germination (www.kegg.jp/kegg/kegg1.html). **B** Heatmap analysis of DEGs involved in photosynthesis, including light reaction and Calvin cycle. The light reaction associated genes photosystem II complex, photosystem II-LHC (light harvesting complex), photosystem I complex, photosystem I-LHC, ATPase subunit, and electron carrier or transfer

Quinoa cotyledons gradually unfolded after the hypocotyl elongation to promote the establishment of green seedlings. Cluster and KEGG enrichment analysis showed that “Photosynthesis proteins”, “Photosynthesis—antenna proteins”, “photosynthesis”, and “Carbon fixation in photosynthetic organisms” were significantly enriched not only in transition of radicle emergence (stage III) to hypocotyl elongation (stage IV) but also in the last cotyledon expensed stage (Fig. 5A). Most genes involved in photosynthesis including light reaction and calvin cycle were induced at the stage of cotyledon expansion (Fig. 5B, Table S3). In this stage post germination, the up-regulated DEGs associating light reaction includes genes encoding the subunit and light harvest of photosystem I and photosystem II respectively, and also several genes are annotated linking to ATP synthase complex and electron carrier or transfer (Fig. 5B, Table S3). Similarly, DEGs annotated as functional components in calvin cycle reaction showed highly expressed at the last stage (Fig. 5B, Table S3). These indicated that subsequent quinoa growth depends not only on the mobilization of storage reserves, but also on photosynthesis. However, the transcript levels of most genes involved in this process exhibited a significant difference between stage III and IV, suggesting the activation of photosynthesis is likely taken place in the stage closely followed radicle emergence. We selected six genes involved in photosynthesis process to do the qRT-PCR analysis, and the substantial increase of these gene in seedling stage comparing stage before (Fig S5), indicating that photosynthesis play a critical role in quinoa post germination and the following green seedling establishment.

### Dynamic metabolism of amino acids happened along with accomplishment of seed germination

**Fig. 6:**
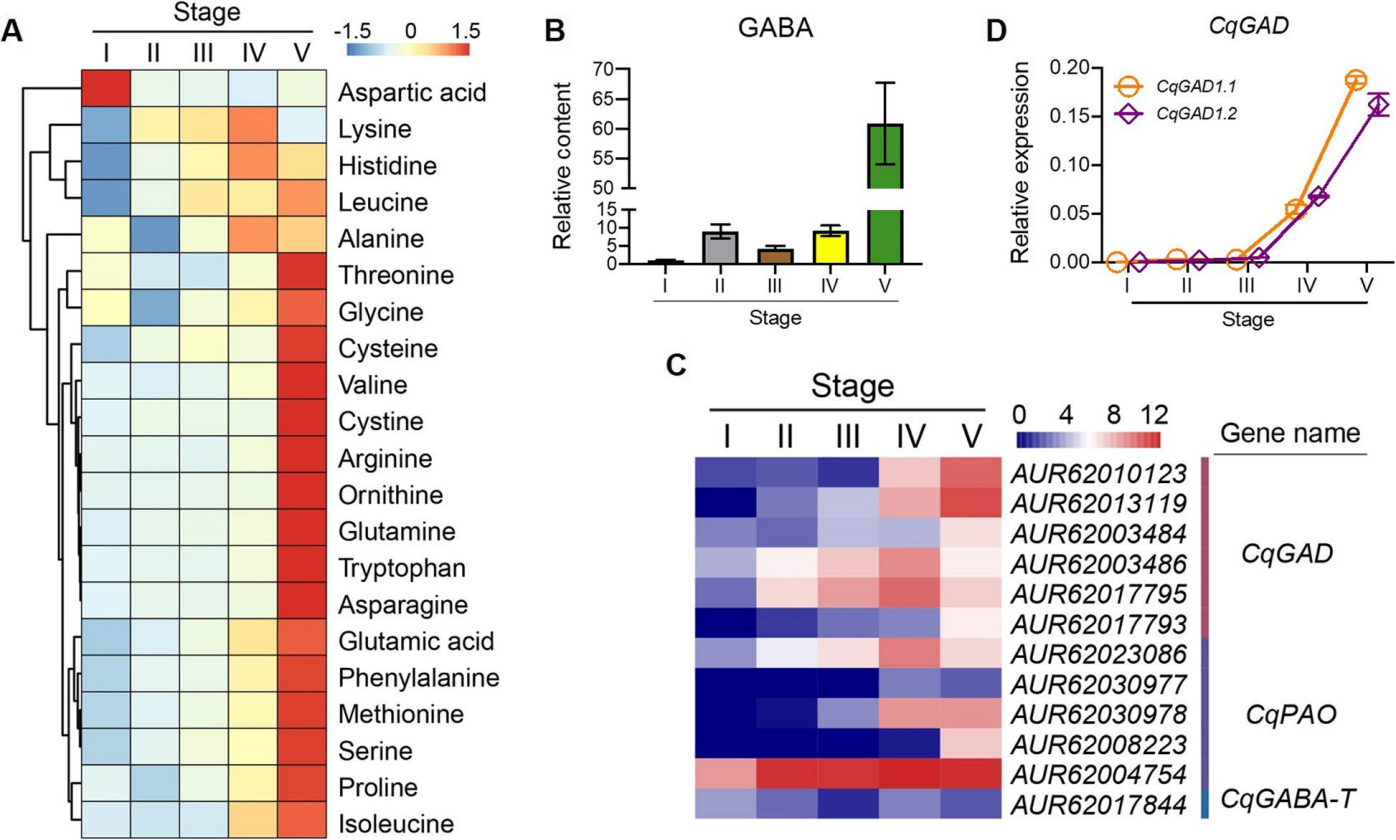
Dynamic metabolism of amino acids happened along with quinoa seed germination. **A** Heatmap analysis showed the contents of different amino acids during transition of quinoa seed germination. **B** Detection of the content of γ-aminobutyric acid (GABA) during quinoa seed germination. Values are means of three independent experiments ± SD, indicated the relative content of target metabolites based on the peak area. **C** Heatmap analysis of genes involved in biosynthesis of GABA. GABA accumulation is associated with the activity of glutamate decarboxylase (GAD), GABA transaminases (GABA-T), glutamate dehydrogenase (GLDH), and polyamine oxidase (PAO). **D** qRT-PCR verified the transcript levels of *CqGAD1.1* and *CqGAD1.2*, which function importantly in GABA biosynthesis. Values are means ± SD (*n* = 3). *** *P* < 0.001, Student's t-test

Amino acids, as one of main metabolic products, serve as important nutritional resources during quinoa germination, provide sufficient raw material for protein synthesis [31]. Quinoa has been recognized as an excellent source of dietary proteins because of the high level of protein and well-balanced amino acids composition, it exhibited relative higher lysine concentration than other cereals [32, 33]. Besides, quinoa is rich in histidine, which is an essentially amino acid for infant [34]. Here, lysine and histidine were significantly accumulated at the stage of hypocotyl elongation. Moreover, most other amino acids content including glycine, alanine, asparagine, glutamine, serine, threonine, cysteine, methionine, isoleucine, leucine, valine, phenylalanine, and tyrosine was significantly increased at the stage of cotyledon expansion, which was necessary for protein biosynthesis during the rapid seedling development (Fig. 6A).

As a non-protein amino acid, γ-aminobutyrate (GABA) was widely involved in signal transduction or regulatory pathways, taking multiple functions under non-stressed and stressed conditions in plants [35, 36]. GABA metabolism through the GABA shunt provides a source for carbon skeletons and energy for down-stream biosynthetic pathways, its accumulation is associated with the activity of glutamate decarboxylase (GAD), GABA transaminases (GABA-T), glutamate dehydrogenase (GLDH), and polyamine oxidase (PAO) [37, 38]. In this study, we first analyzed the content of GABA and found it was significantly accumulated in the final designated stage, while it showed not different in the early stages (Fig. 6B). Then we analyzed the DEGs associating with GABA metabolism and found 10 genes including *CqGAD*, *CqGABA-T*, and *CqPAO*, which were significantly up-regulated at the stage of hypocotyl elongation (Fig. 6C, Table S3). Subsequently, two genes annotated as *CqGAD1.1* (*AUR62013119*) and *CqGAD1.2* (*AUR62010123*) were chosen for expression verification, which were highly expressed at the stages of hypocotyl elongation and cotyledon expansion (Fig. 6D). The result suggested that GABA may play an important role in the regulation of quinoa germination and the transition to seedling establishment.

## Conclusion

In this study towards quinoa seed germination, we used transcriptomic and metabolomic approaches to analyze the dynamic physiological processes and the corresponding regulatory network at five distinct stages from dry seeds to green seedlings, including dry seed, imbibed seed, radical emergence, hypocotyl elongation, and cotyledon expansion. Genes associating with phytohormones synthesis and signal transduction, energy reserves mobilization, cell wall remodeling, photosynthesis, amino acids metabolism, and GABA synthesis during seed germination to seedling establishment were characterized. Correlation analysis of transcriptome and metabolic profiling showed that phytohormones signals play key roles in early quinoa seed germination, and ABA plays relatively conserved roles in inhibiting the germination of quinoa seeds like other plant species. Starch and sucrose metabolism provided the main energy at the very early stages during quinoa seed germination. We observed significantly higher expression of starch and sucrose metabolism-related genes and greater accumulation of related metabolites. Cell-wall remodeling and modification associating processes were enriched in the intervals of dry seeds versus seeds imbibition, and is crucial for the fast growth phase such as seed germination. Photosynthesis process was highly activated after the stage of hypocotyl elongation, play an important role in quinoa post germination and the following green seedling establishment. GABA was accumulated in the post-germination stage, and may participated in the regulation of quinoa germination and the transition to seedling establishment.

Quinoa is featured with its comprehensive nutrition and high stress tolerance, making it a potential new stable food with high protein content [39, 40]. However, quinoa germination was strongly influenced by the environment. Quinoa preharvest sprouting is a problem limiting its extensive cultivation [41]. At present, research on quinoa seed germination and its regulation is limited. Seed germination is an essential step in the initiation of a new life cycle in plants with complex changes at the transcript and metabolic levels. The single-omic approach is actually not appropriate for clarifying complex biological phenotypes. This study is the first comprehensive analysis of transcriptome and metabolome profile in germinating quinoa seeds, providing the experimental data for in-depth study of regulatory mechanisms associated with quinoa seed germination. The multi-omics analysis revealed global changes in metabolic pathways and phenotype during and post quinoa seed germination, paving ways to solve the problems of quinoa preharvest sprouting and seed vigor maintenance and serving as an important resource for study of gene functions and quinoa improvement.

## Materials and methods

### Plant material and growth condition

Quinoa seeds of the variety "Qaidam Red-1", which was granted by the Qinghai Provincial Crop Variety Examination and Approval Committee with the certification number: Qinghai Identification Record 2,018,002, were obtained from the Institute of Crop Breeding and Cultivation, Qinghai Academy of Agriculture and Forestry Sciences (ICBC-QAAFS). Mature seeds were collected from the experimental field of this institute located in Ulan County, Qinghai Province, and identified by associate professor Youhua Yao from ICBC-QAAFS. Collection of quinoa seeds complies with institutional and national guidelines. The seeds were placed in 12 cm × 12 cm culture plates for germination assay. Before germinating, the seeds were sterilized with 10% 84 disinfectants for 10 min, and then washed with sterile water six times with each for 5 min. The culture plates were placed into a growth chamber at 24 °C with 16 h light / 8 h dark. Germination assay was performed with three biological replicates. The fresh weight of ten seeds was measured at different germination and growth time. Samples were harvested at different stages including dry seed, imbibed seed, radical emergence, hypocotyl elongation, and cotyledon expansion, and then put into liquid nitrogen for further analysis.

### RNA extraction and quantitative real-time PCR (qRT-PCR)

The total RNA was extracted from collected samples using TRIzol RNA isolation reagent (Sigma-Aldrich, MO, USA) following the manufacturer’s instructions for further transcriptome sequencing. For qRT-PCR assay, the isolated total RNA was treated by DNase I to remove the genomic DNA, and cDNA was synthesized using a cDNA Synthesis SuperMix Kit (TransGen Biotech, China). qRT-PCR was performed on ABI 7500 Fast Real-Time System (Applied Biosystems, USA) using a TransStart Top Green qPCR Supermix (TransGen Biotech, China) to verify the transcript levels of target genes involved in quinoa germination, and *CqMON* gene was used as the internal control to calculate the genes expression by the 2^–ΔΔCt^ method [42]. Each qRT-PCR analysis was repeated for three times with three biological replicates, and gene-specific primers were showed in Table S4.

### Transcriptome sequencing and analysis

Three replicated samples for each stage were used for total RNA extraction and further transcriptome sequencing. By using NEBNEXT® UltraTM RNA Library Prep Kit for ILLumina® (NEB, USA) following manufacturer’s instructions, the libraries were constructed and sequenced using illumine Hiseq 4500 platform to produce 150-bp pair-end raw reads. Using Fastp [43], the raw data were filtered by removing the sequencing adaptor and reads with low quality, and trimming 20 bp in front of the raw reads to obtain clean reads. Then clean reads were aligned to the *Chenopodium quinoa* reference genome (Cquinoa_392_v1.0) [14] by HISAT (hierarchical indexing for spliced alignment of transcripts) [44]. With these mapped reads in each sample, we used featureCounts [45] to calculate the raw read counts of each gene in each sample. We normalized and identified different expression of genes between different stages of gemination by DEseq2 [46], which are a R packages with a model upon the negative binomial distribution and adjust *P*-values by utilizing the Benjamini and Hochberg's approach to control the false discovery rate (FDR). Applying the method, genes with an adjusted *P*-value (padj) < 0.05 and | Log_2_ (fold change) |≥ 2 were as significantly differential expression (DEGs) between two samples. In addition, we annotated the DEGs with genome annotation files “Cquinoa_392_v1.0.annotation_info.txt” from the JGI (Joint Genome Institute) databases. The functional enrichment analysis including GO [47] and KEGG [48] for DEGs were carried out via the clusterProfiler R package [49].

### Sample preparation and metabolite data acquisition for GC–MS analysis

The seeds were harvested at different germination and growth stages, and ground into powder with liquid nitrogen. Then were soaked in extraction buffer (Acetonitrile / Isopropanol / Water, 3/3/2), mixed with 5 μL of Myristic acid-d27, vortexed for 1 min at 2000 rpm, followed by ultrasonic extraction for 32 s. After 5 min of centrifugation at 13,000 rpm, the supernatant was dried with nitrogen gas. The samples were then derivatized with 20 μL of 40 mg/mL methoxyamine hydrochloride/pyridine, 30 °C incubation for 90 min. Thereafter mixed with 90 μL of 1% TMCS (trimethylchlorosilane) in MSTFA (N-methyl-N-(trimethylsilyl) trifluoroacetamide) and incubation for 30 min at 37 °C, after centrifugation for 5 min (13,000 rpm, 4 °C), the supernatant was collected and stored at -20 °C until GC–MS analysis. In addition, the equivalent volume of prepared samples was blended into a large sample, and then divided into eight quality control samples for monitoring instrument’s precision and stability.

Metabolite profiling was conducted using the GC–MS (Agilent Technologies, Glostrup, Denmark) with three replicates. The GC–MS system contains an Agilent 7890A gas chromatograph and an Agilent 5975C series mass spectrometric detector. Gas chromatograph separation was performed on an Agilent DB5-MS column (30 m × 250 μm × 0.25 μm). Raw GC–MS data was transformed into NetCDF format through Agilent Chrom Station software (Agilent Technologies, USA). Erah package was used for data preprocessing such as peak identification, retention time alignment, automatic integration, etc. Compared with the standardized information in GMD database, the mass spectrograms with high similarity and high match of retention index were selected, metabolites were identified. Principal component analysis (PCA) and one-way ANOVA analysis were performed to observe the differences in metabolic composition among different stages. The adjusted *P-*value (padj) less than 0.05 was considered statistically different.

### Detection of ABA and starch content in quinoa germinating seeds

The quinoa samples were grinded using liquid nitrogen and then dissolved in PBS buffer (pH7.4). After centrifuging at 3000 r/min for 20 min, the supernatant was collected and stored for further analysis. ABA content was measured using the Plant Hormone Abscisic acid Elisa kit (MLBIO Biotechnology Co.Ltd, China, Shanghai) with three biological replicates. ABA standard was diluted to a series concentration of 0, 5, 10, 20, 40, and 80 ng/mL. The samples were incubated on the coating plate with enzyme-labeled reagent for 60 min, and then washed with PBST buffer for five times. Absorbance was read at 450 nm using a Synergy microplate fluorescence reader (Bio-Tek Instruments Inc., Winooski, VT, USA). Starch content was measured using the amyloglucosidase / α-amylase method (Total starch assay procedure, Megazyme, Ireland) according to the manufacturer’s instructions. Sprouted quinoa was freeze-dried and ground to 0.5 mm screen. Distilled water was used as the control group. The absorbance of resulting solution at 510 nm was read using a Synergy microplate fluorescence reader. Starch content detection was carried out with three biological replicates.

## Supplementary Information

**Additional file 1** **Fig. S1.** Analysis of fresh weight changes and characterization of transcriptomic data. **A** The fresh weight changes of germinating quinoa seeds. **B** Venn diagram of differentially expressed genes (DEGs) in quinoa germinating seeds at the different stage intervals. **C** KEGG analysis of DEGs identified during different stages of quinoa seed germination (www.kegg.jp/kegg/kegg1.html). **Fig. S2** Venn diagram and KEGG of differential metabolites identified by GC-MS. **A** Venn diagram of differentially metabolites in quinoa germinating seeds at the different stage intervals. **B** KEGG analysis of differentially metabolites identified during quinoa seed germination. **Fig. S3**. Biosynthesis and signaling transduction of different phytohormones participated in seed germination in the early stage. Heatmap analysis of DEGs involved in biosynthesis and signaling transduction of gibberellin (A), auxin (B), cytokinin (C) *GA*_*20*_*OX*, *GIBBERELLIN 20−OXIDASE*. *GA*_*2*_*OX*, *IAA4*, *INDOLE-3-ACETIC ACID INDUCIBLE 4*. *GH3*, *GRETCHEN HAGEN 3*. *SAUR*, *SMALL AUXIN UPREGULATED RNA*. *IPT*, *ISOPENTENYLTRANSFERASE*. *CKX*, *CYTOKININ OXIDASE*. *ARR*, *ARABIDOPSIS RESPONSE REGULATOR*. **Fig. S4** Gene ontology analysis reveals cell-wall remodeling process was activated in early stage of quinoa seed germination. GO analysis of DEGs among dry seeds (stage I) versus seeds imbibition (stage II). GO enrichment terms were classified into three functional groups including cellular components, molecular functions and biological process. **Fig. S5** qRT-PCR verification of the genes involved in photosynthesis process. The transcript levels of four genes involved in photosynthesis, *CqPsaK *(photosystem I subunit), *CqLHCA1* and *CqLHCA3 *(photosystem I-LHC), *CqLHCA4.2 *(photosystem II-LHC), *CqPETC* (electron transfer) and *CqRBCS1A *(Calvin-cycle related), were detected by qRT-PCR assay, which were upregulated at imbibed stage during germination. Values are means ± SD (*n* = 3). *** *P* < 0.001, Student's t-test

**Additional file 2** **Table S1** Library read analyses statistics of the transcriptom from five stages.

**Additional file 3** **Table S2** DEGs between the stages.

**Additional file 4 Table S3** Expression of DEGs in the heatmap analysis.

**Additional file 5** **Table S4** Primers and sequences used in qRT-PCR analysis

## Data Availability

The raw reads generated during the current study have been deposited in BioProject with the accession number ofPRJNA826741(https://www.ncbi.nlm.nih.gov/bioproject/PRJNA826741).
