# Supplementary material for: Transcriptomic and metabolomic landscape of quinoa during seed germination
Source: BMC Plant Biol. 2022 May 10;22:237. doi: 10.1186/s12870-022-03621-w (PMC9088103; doi:10.1186/s12870-022-03621-w)
Supplement: Supplementary file 1 — Additional file 1 Fig. S1. Analysis of fresh weight changes and characterization of transcriptomic data. A The fresh weight changes of germinating quinoa seeds. B Venn diagram of differentially expressed genes (DEGs) in quinoa germinating seeds at the different stage intervals. C KEGG analysis of DEGs identified during different stages of quinoa seed germination (www.kegg.jp/kegg/kegg1.html). Fig. S2 Venn diagram and KEGG of differential metabolites identified by GC-MS. A Venn diagram of differentially metabolites in quinoa germinating seeds at the different stage intervals. B KEGG analysis of differentially metabolites identified during quinoa seed germination. Fig. S3. Biosynthesis and signaling transduction of different phytohormones participated in seed germination in the early stage. Heatmap analysis of DEGs involved in biosynthesis and signaling transduction of gibberellin (A), auxin (B), cytokinin (C) GA20OX, GIBBERELLIN 20−OXIDASE. GA2OX, IAA4, INDOLE-3-ACETIC ACID INDUCIBLE 4. GH3, GRETCHEN HAGEN 3. SAUR, SMALL AUXIN UPREGULATED RNA. IPT, ISOPENTENYLTRANSFERASE. CKX, CYTOKININ OXIDASE. ARR, ARABIDOPSIS RESPONSE REGULATOR. Fig. S4 Gene ontology analysis reveals cell-wall remodeling process was activated in early stage of quinoa seed germination. GO analysis of DEGs among dry seeds (stage I) versus seeds imbibition (stage II). GO enrichment terms were classified into three functional groups including cellular components, molecular functions and biological process. Fig. S5 qRT-PCR verification of the genes involved in photosynthesis process. The transcript levels of four genes involved in photosynthesis, CqPsaK (photosystem I subunit), CqLHCA1 and CqLHCA3 (photosystem I-LHC), CqLHCA4.2 (photosystem II-LHC), CqPETC (electron transfer) and CqRBCS1A (Calvin-cycle related), were detected by qRT-PCR assay, which were upregulated at imbibed stage during germination. Values are means ± SD (n = 3). *** P < 0.001, Student's t-test [file 12870_2022_3621_MOESM1_ESM.docx]

Figure S1


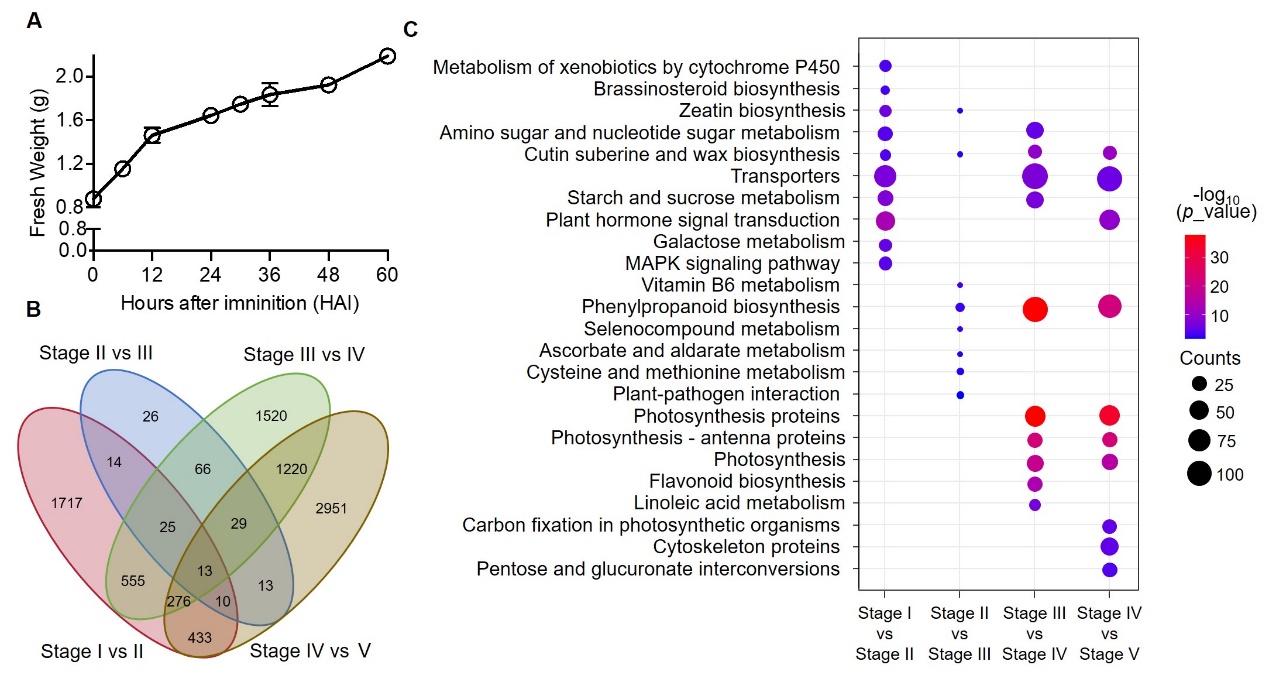


**Figure S1. Analysis of fresh weight changes and characterization of transcriptomic data**.

A, The fresh weight changes of germinating quinoa seeds. B, Venn diagram of differentially expressed genes (DEGs) in quinoa germinating seeds at the different stage intervals. C, KEGG analysis of DEGs identified during different stages of quinoa seed germination ([www.kegg.jp/kegg/kegg1.html](http://www.kegg.jp/kegg/kegg1.html)).

Figure S2


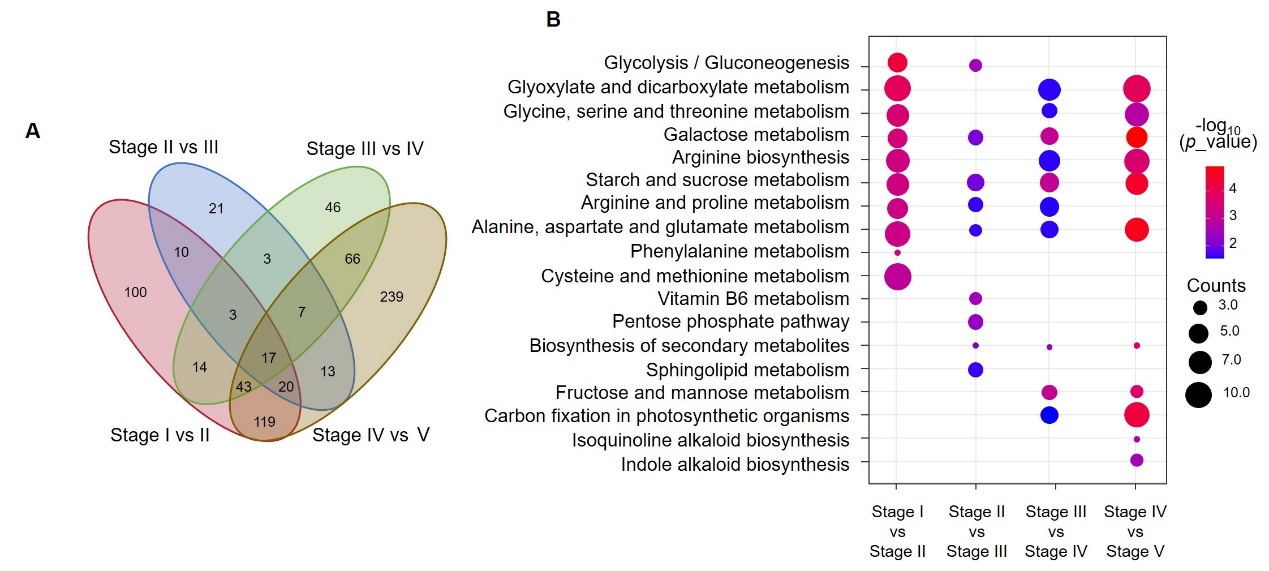


**Figure S2. Venn diagram and KEGG of differential metabolites identified by GC-MS**.

A, Venn diagram of differentially metabolites in quinoa germinating seeds at the different stage intervals. B, KEGG analysis of differentially metabolites identified during quinoa seed germination.

Figure S3


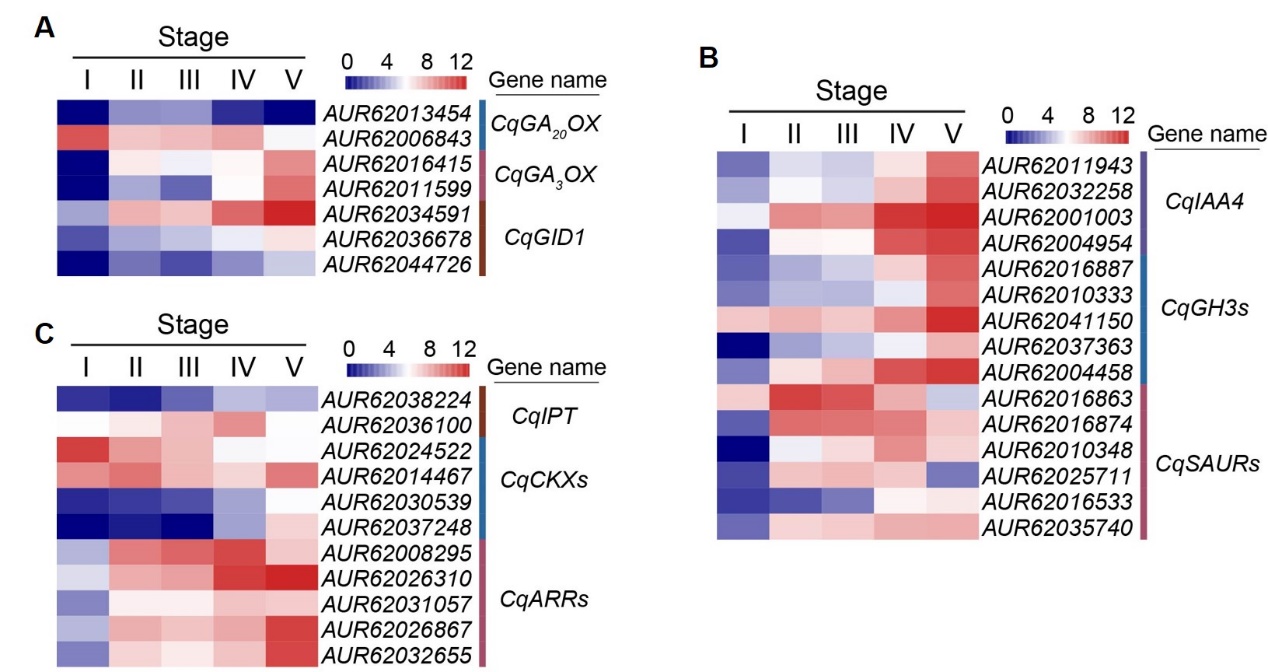


**Figure S3**. **Biosynthesis and signaling transduction of different phytohormones participated in seed germination in the early stage.**

Heatmap analysis of DEGs involved in biosynthesis and signaling transduction of gibberellin (A), auxin (B), cytokinin (C). *GA_20_OX*, *GIBBERELLIN 20−OXIDASE*. *GA_2_OX*, *GIBBERELLIN 2−OXIDASE*. *GID*, *GIBBERELLIN-INSENSITIVE DWARF 1*. *IAA4*, *INDOLE-3-ACETIC ACID INDUCIBLE 4*. *GH3*, *GRETCHEN HAGEN 3*. *SAUR*, *SMALL AUXIN UPREGULATED RNA*. *IPT*, *ISOPENTENYLTRANSFERASE*. *CKX*, *CYTOKININ OXIDASE*. *ARR*, *ARABIDOPSIS RESPONSE REGULATOR.*

Figure S4


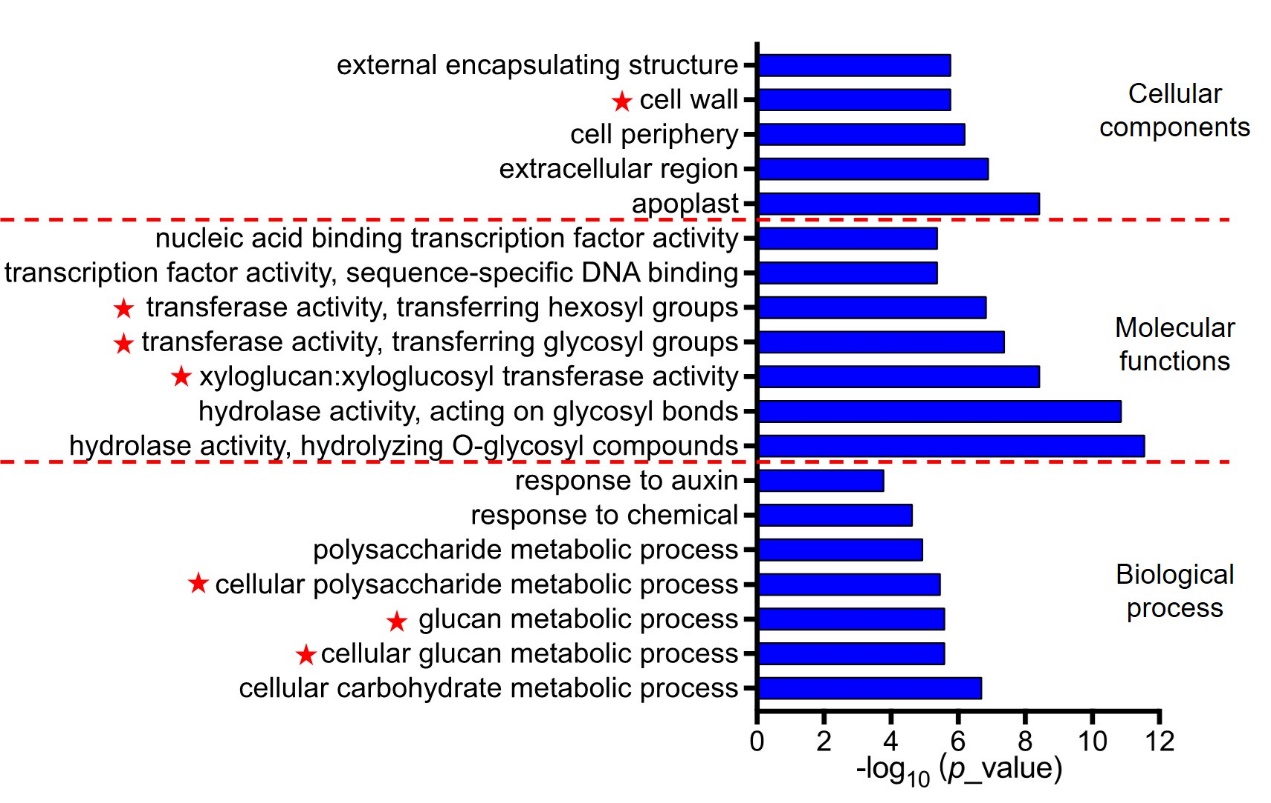


**Figure S4. Gene ontology analysis reveals cell-wall remodeling process was activated in early stage of quinoa seed germination.**

GO analysis of DEGs among dry seeds (stage I) versus seeds imbibition (stage II). GO enrichment terms were classified into three functional groups including cellular components, molecular functions and biological process.

Figure S5


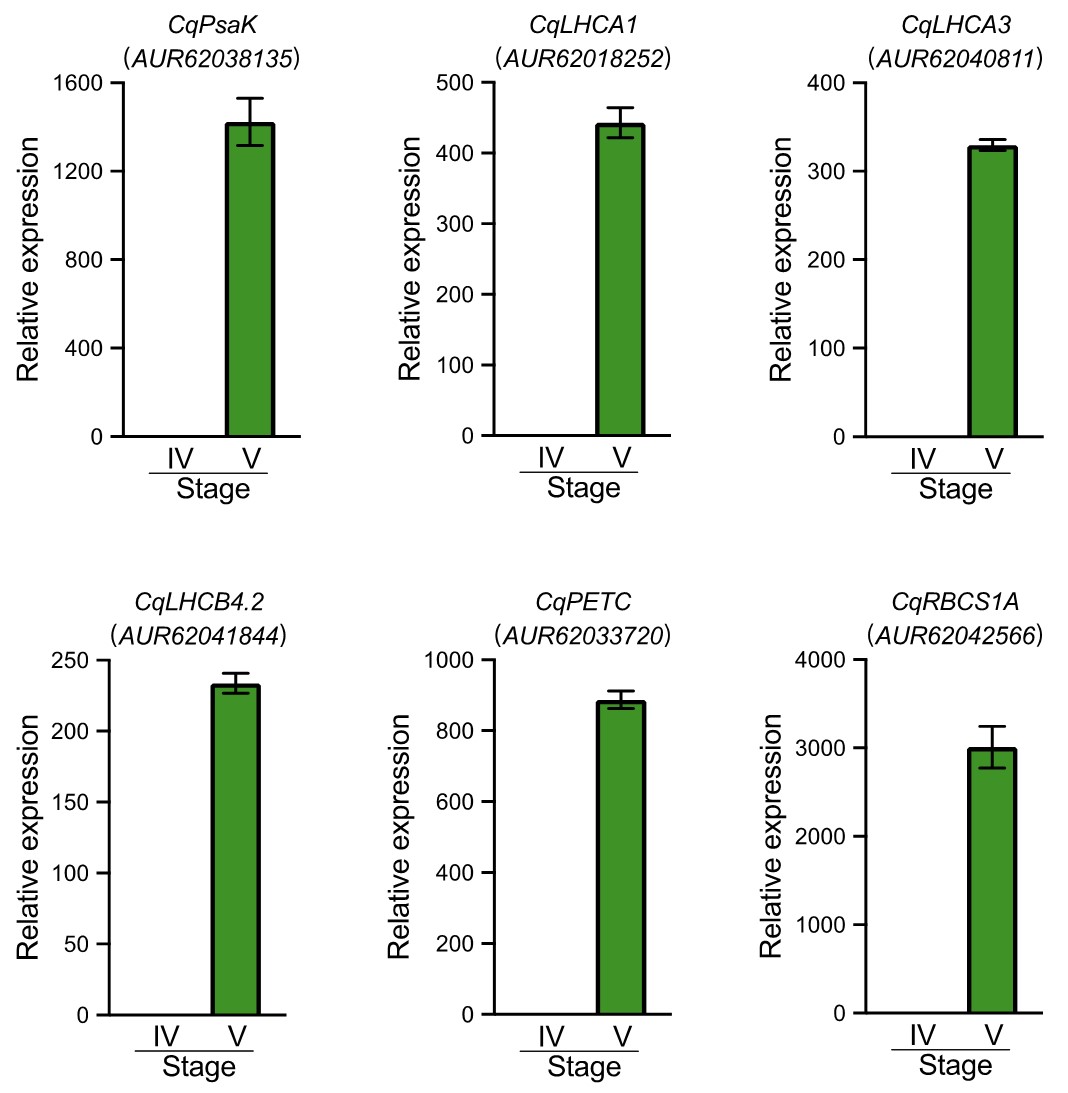


**Figure S5. qRT-PCR verification of the genes involved in photosynthesis process.**

The transcript levels of four genes involved in photosynthesis, *CqPsaK* (photosystem I subunit), *CqLHCA1* and *CqLHCA3* (photosystem I-LHC), *CqLHCA4.2* (photosystem II-LHC), *CqPETC* (electron transfer) and *CqRBCS1A* (Calvin-cycle related), were detected by qRT-PCR assay, which were upregulated at imbibed stage during germination. Values are means ± SD (n = 3). *** *P* < 0.001, Student's t-test.
